# The Influence of Seasonality on Secondary Metabolite Profiles and Neuroprotective Activities of Moss *Hypnum cupressiforme* Extracts: In Vitro and In Silico Study

**DOI:** 10.3390/plants11010123

**Published:** 2022-01-01

**Authors:** Tanja M. Lunić, Marija R. Mandić, Mariana M. Oalđe Pavlović, Aneta D. Sabovljević, Marko S. Sabovljević, Biljana Đ. Božić Nedeljković, Bojan Đ. Božić

**Affiliations:** 1 Institute of Physiology and Biochemistry “Ivan Đaja”, Faculty of Biology, University of Belgrade, 11000 Belgrade, Serbia; lunictanja@hotmail.com (T.M.L.); mandicmarija5@gmail.com (M.R.M.); 2 Institute of Botany and Botanical Garden “Jevremovac”, Faculty of Biology, University of Belgrade, 11000 Belgrade, Serbia; marianao@bio.bg.ac.rs (M.M.O.P.); aneta@bio.bg.ac.rs (A.D.S.); marko@bio.bg.ac.rs (M.S.S.); 3 Department of Botany, Institute of Biology and Ecology, Faculty of Science, Pavol Jozef Šafárik University in Kosice, Mánesova 23, 040 01 Kosice, Slovakia

**Keywords:** bryophytes, moss extract, *Hypnum cupressiforme*, seasonal changes, secondary metabolites, anti-inflammatory, anti-neurodegenerative, neuroprotection, molecular docking

## Abstract

Numerous representatives of mosses, including *Hypnum cupressiforme*, have been used to alleviate different inflammation-related conditions. However, the mode of action underlying this anti-inflammatory potential has been poorly understood. Moreover, the influence of seasonality on the chemical composition and biological activity of mosses is generally overlooked. This study aimed to investigate the influence of seasonal changes (spring, summer, and autumn) on secondary metabolite composition and biological activities of ethyl acetate *H. cupressiforme* extracts. Antioxidant activity was measured using β-carotene bleaching assay, while MTT, NBT, ELISA, and Griess assays were carried out to explore the anti-neuroinflammatory and neuroprotective potential of extracts. Inhibitory activities on acetylcholinesterase and tyrosinase were assessed experimentally and by docking analysis. The highest content of secondary metabolites and antioxidant activity were observed in moss during the summer. Extracts inhibited the secretion of ROS, NO, TNF-α, and IL-6, alleviating the inflammatory potential of H_2_O_2_ and LPS in microglial and neuronal cells. Strong inhibitory effects on acetylcholinesterase and tyrosinase were observed in vitro. Docking analyses revealed high-affinity interactions of secondary metabolites present in *H. cupressiforme* with important enzyme residues. Altogether, these results reveal the neuroprotective potential and the significance of seasonal fluctuations on secondary metabolite content and biological activities in moss *H. cupressiforme*.

## 1. Introduction

Mosses are small, non-vascular plants that belong to the second-largest group of terrestrial plants, bryophytes [1]. They occur in a wide range of habitats and form an essential component of numerous ecosystems, thus contributing to their proper functioning. Among other things, mosses have found application in biomonitoring of air pollutants and ethnopharmacology [2,3]. They have been used in traditional medicine for the treatment of skin infections, wounds, and burns, as well as different inflammation-related conditions (fever, adenotonsillitis, rhinitis, and pneumonia) [3]. Moreover, mosses exhibit well-known antitumor, antimicrobial, and antifungal activities [4,5,6]. In the present study, we have focused on *Hypnum cupressiforme* Hedw., a pleurocarpous moss species. This moss can be found in nearly all continents and climate zones, while in Serbia it is one of the most common moss species [7,8]. Despite the prevalence of this moss, studies regarding *H. cupressiforme*’s chemical composition and biological activities are rare, while biological assays usually involve the analysis of its antioxidant, antimicrobial, and antitumor potential [9,10]. 

Nowadays, a great biological interest is brought to bryophytes since they contain a broad spectrum of secondary metabolites, which are produced to combat different biotic and abiotic stress (microorganisms, insects, UV radiation, and different environmental conditions). The majority of stress factors have a clear seasonal pattern, which implies that the total content and relative proportions of secondary metabolites in plants oscillate depending on seasonal i.e., environmental changes. Consequently, these changes influence the therapeutic efficacy of the particular plant, as well. Several studies have been conducted to investigate the time and season of harvesting different medicinal plants and their parts [11]. However, when it comes to bryophytes, only a few studies analyzed the chemical and biochemical responses of bryophytes to seasonal changes [12,13,14]. For instance, a study about the lipid composition in *Sphagnum* mosses revealed a clear seasonal pattern in the total lipid content of this moss [13]. A different study investigated the chemical composition and concentration of particular metabolites in four different moss species during the seasons and found seasonal variations within the major groups of moss secondary metabolites [12]. Additionally, a study about different liverwort species has shown seasonal variation in total phenolic and flavonoid content, as well as in antioxidant and polyphenol oxidase enzymes [14]. Since there is no general rule about the harvesting time for better yield and ratio of specific secondary metabolites [11], studies regarding the seasonal variation of moss secondary metabolites and biological potential are useful to find the optimal conditions for harvesting of a particular moss species. 

In our recently published paper [10], the ethyl acetate extract of *H. cupressiforme* exhibited a significant anti-neuroinflammatory activity by reducing the production of NO in lipopolysaccharide (LPS)-stimulated BV2 microglial cells, along with potent activities to inhibit tyrosinase and acetylcholinesterase (AChE). LPS-induced stimulation of microglial cells is a well-known and extensively employed model for the study of neuroinflammation, which underlies the pathogenesis of neurological disorders such as Alzheimer’s (AD) and Parkinson’s disease (PD) [15]. Activated microglial cells release diverse pro-inflammatory cytokines and neurotoxic mediators including nitric oxide (NO), tumor necrosis factor-alpha (TNF-α), interleukin-1β (IL-1β), interleukin-6 (IL-6), and reactive oxygen species (ROS) [16]. The accumulation of these factors inevitably leads to inflammation and consequent neuronal damage. Therefore, the inhibition of excessive and prolonged microglial activation may alleviate neurodegeneration. Additionally, the inhibition of enzymes, such as AChE and tyrosinase, involved in the pathogenesis of AD and PD represents a promising pathway in the treatment of these neurological disorders [10]. 

Based on our previously published data [10], the next objective was to investigate whether the change of season has an impact on the secondary metabolite composition and biological activities of moss *H. cupressiforme*. For this purpose, five selected classes of secondary metabolites, as well as antioxidant, anti-neuroinflammatory, anti-neurodegenerative, and neuroprotective potential of moss *H. cupressiforme*, have been examined. Finally, docking studies have been performed with the aim to identify the extract constituents from *H. cupressiforme*, which contribute the most to the corresponding anti-neurodegenerative effect.

## 2. Results

### 2.1. (Bio)Chemical Evaluation 

**Figure 1:**
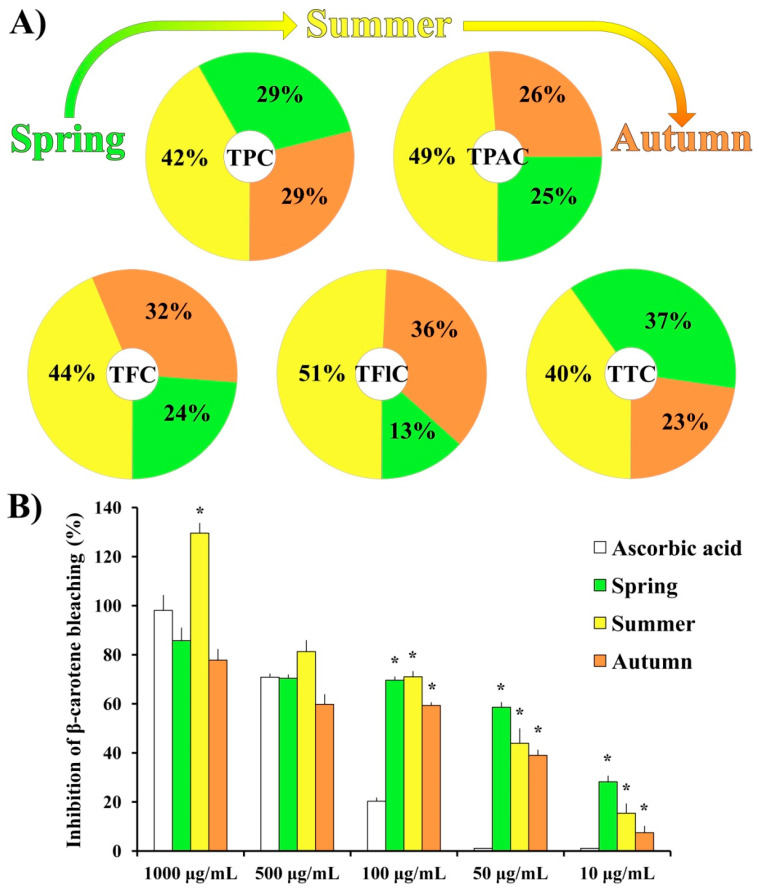
(**A**) Chemical composition and (**B**) antioxidant activity of *H. cupressiforme* ethyl acetate extracts from different seasonal aspects (spring, summer, and autumn). The results are presented as the mean ± standard error (* *p* < 0.05 different seasons vs. standard substance ascorbic acid).

The relative ratios of total phenolic content (TPC), total phenolic acid content (TPAC), total flavonoid content (TFC), total flavonol content (TFlC), and total triterpenoid content (TTC) for three seasonal aspects (spring [10], summer, and autumn) of moss *H. cupressiforme* ethyl acetate extracts are presented in Figure 1A. The absolute values with corresponding statistics are given in Table S1. It should be noted that the data obtained for the spring aspect, although already published in our previous study [10], are also presented in Table S1, together with summer and autumn results in order to compare the chemical composition of moss extracts from three different investigated seasons.

As shown in Figure 1A, the chemical analysis of *H. cupressiforme* ethyl acetate extracts revealed an unequal distribution of secondary metabolites among different seasonal aspects. The highest concentrations of all investigated secondary metabolites were found in the summer aspect and they were significantly higher compared to both spring and autumn seasons (Table S1). The only exception was TTC, where the summer aspect of the moss was statistically significant regarding the autumn aspect only.

The results of the β-carotene bleaching assay (Figure 1B) showed that, at the lower tested concentrations (100, 50, and 10 μg/mL), investigated extracts from all seasonal aspects exhibited significantly higher activity than standard natural antioxidant—ascorbic acid. Moreover, at the highest applied concentration (1000 μg/mL), extract from the summer aspect exhibited a significantly better inhibition rate in comparison to the same concentration of the standard substance (129.6% for the summer season vs. 98.1% for the AA). Calculated IC50 values for ascorbic acid, spring, summer, and autumn aspects are as follows: 250.88 μg/mL; 54.45 μg/mL; 36.01 μg/mL; 63.53 μg/mL, respectively. 

### 2.2. Anti-Neuroinflammatory Potential of H. cupressiforme Extracts

#### 2.2.1. H_2_O_2_-Stimulated BV2 Microglial Cells

**Figure 2:**
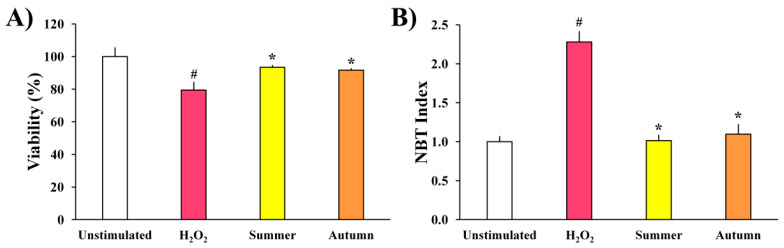
The effects of *H. cupressiforme* ethyl acetate extracts from different seasonal aspects (summer and autumn) on (**A**) metabolic activity and (**B**) ROS production in H_2_O_2_-stimulated BV2 microglial cells. The results are presented as the mean ± standard error (# *p* < 0.05 unstimulated cells vs. H_2_O_2_-stimulated; * *p* < 0.05 H_2_O_2_-stimulated vs. moss-treated and H_2_O_2_-stimulated cells).

Extracts of moss *H. cupressiforme* from summer and autumn seasons were evaluated for their effects on the metabolic activity and ROS production of H_2_O_2_-stimulated murine BV2 microglial cells, and the results are presented in Figure 2.

As shown in Figure 2A, the metabolic activity of BV2 cells treated only with H_2_O_2_ was significantly reduced when compared to the control, unstimulated cells. However, after simultaneous treatment with H_2_O_2_ and extracts, a significant recovery of metabolic activity of BV2 cells was detected, in comparison to the cells treated only with H_2_O_2_. Extracts from both seasons exhibited similar activities and did not differ statistically.

As expected, in comparison to unstimulated cells, the production of ROS by BV2 cells increased after stimulation with H_2_O_2_ (Figure 2B). However, investigated extracts significantly reduced ROS production (H_2_O_2_-induced), expressed as NBT index, from 2.3 to 1 and 1.1 (summer and autumn aspects, respectively) bringing them to the level of unstimulated cells. These results indicate that the investigated extracts possess a strong and significant antioxidant potential in the investigated cell model.

#### 2.2.2. LPS-Stimulated BV2 Microglial Cells

**Figure 3:**
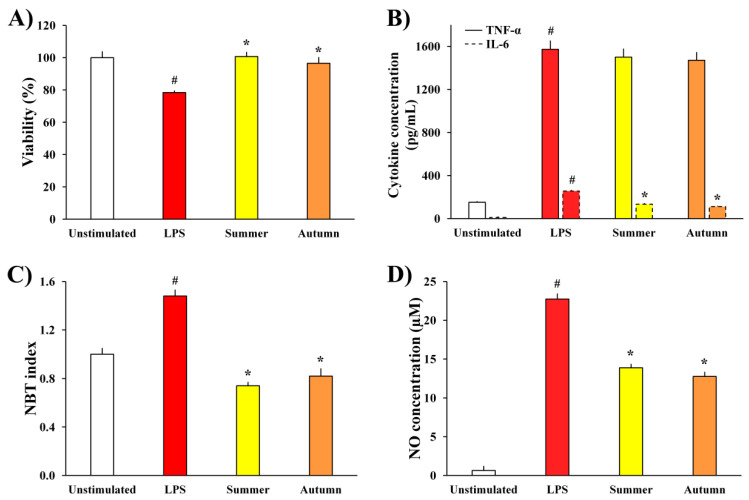
The effects of *H. cupressiforme* ethyl acetate extracts from different seasonal aspects (summer and autumn) on (**A**) metabolic activity, (**B**) cytokine production, (**C**) ROS production, and (**D**) NO production by LPS-stimulated BV2 microglial cells. The results are presented as the mean ± standard error (# *p* < 0.05 unstimulated cells vs. LPS-stimulated; * *p* < 0.05 LPS-stimulated vs. moss-treated and LPS-stimulated cells).

The influence of moss extracts on LPS-stimulated BV2 cells was determined by measuring the metabolic activity of the cells, production of pro-inflammatory cytokines (TNF-α and IL-6) and pro-inflammatory mediators (ROS and NO), and the results are presented in Figure 3.

The data presented in Figure 3A show that the metabolic activity of LPS-treated BV2 cells was significantly reduced compared to unstimulated cells, as a consequence of LPS stimulation. Simultaneous treatment of cells with LPS and extracts of moss *H. cupressiforme* from summer and autumn seasons normalized the metabolic activity of LPS-stimulated BV2 cells, thus leading to their full recovery. 

Extracts from summer and autumn seasons were further evaluated for their effects on the production of inflammatory cytokines, TNF-α and IL-6, by BV2 microglial cells. As it is presented in Figure 3B, LPS stimulation significantly increased the levels of both cytokines in the cell supernatants, while treatment with moss extracts significantly reduced the levels of IL-6 compared to the level in supernatants of only LPS-treated control cells. On the other hand, although the TNF-α production was not significantly reduced by treatment with *H. cupressiforme*, it was diminished by a certain percentage (4.5% for summer and 6.5% for autumn season).

Moreover, as a response to LPS stimulation, there was a significant increase in the production of inflammatory molecules (ROS and NO, Figure 3C,D, respectively) in comparison to unstimulated cells. Nevertheless, investigated moss extracts significantly diminished the production of ROS and NO, bringing them to the level of non-stimulated controls and thus reducing inflammation.

### 2.3. Neuroprotective Potential of H. cupressiforme Extracts

**Figure 4:**
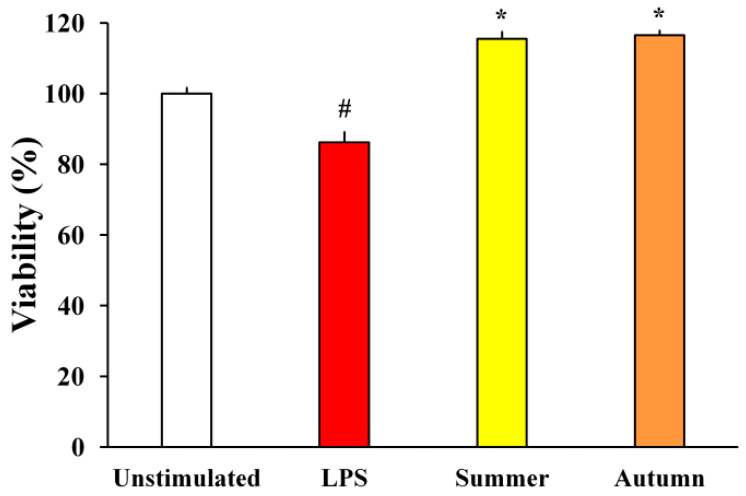
The effects of LPS-stimulated and moss-treated supernatants of BV2 cells on SH-SY5Y metabolic activity. The results are presented as the mean ± standard error (# *p* < 0.05 unstimulated cells vs. LPS-stimulated; * *p* < 0.05 LPS-stimulated vs. moss-treated and LPS-stimulated cells).

With the aim to determine whether moss extracts can inhibit the neuronal death caused by LPS-induced BV2 neurotoxicity, the metabolic activity of SH-SY5Y neurons was assessed. The influence of BV2 supernatants treated with LPS and moss extracts on SH-SY5Y neurons is shown in Figure 4.

The results presented in Figure 4 show that supernatants of BV2 cells treated only with LPS have led to a reduction in the metabolic activity of SH-SY5Y cells. On the other hand, the metabolic activity of SH-SY5Y treated with supernatants of BV2 cells simultaneously treated with LPS and moss extracts was significantly higher. Supernatants of BV2 cells treated with moss extracts increased SH-SY5Y cell viability by 29.3% (summer aspects) and 30.3% (autumn aspect) compared to media collected from cells treated only with LPS. This data reveals that moss extracts can provide neuroprotection of SH-SY5Y neurons induced by microglia-mediated LPS neurotoxicity.

### 2.4. Anti-Neurodegenerative Potential of H. cupressiforme Extracts

**Figure 5:**
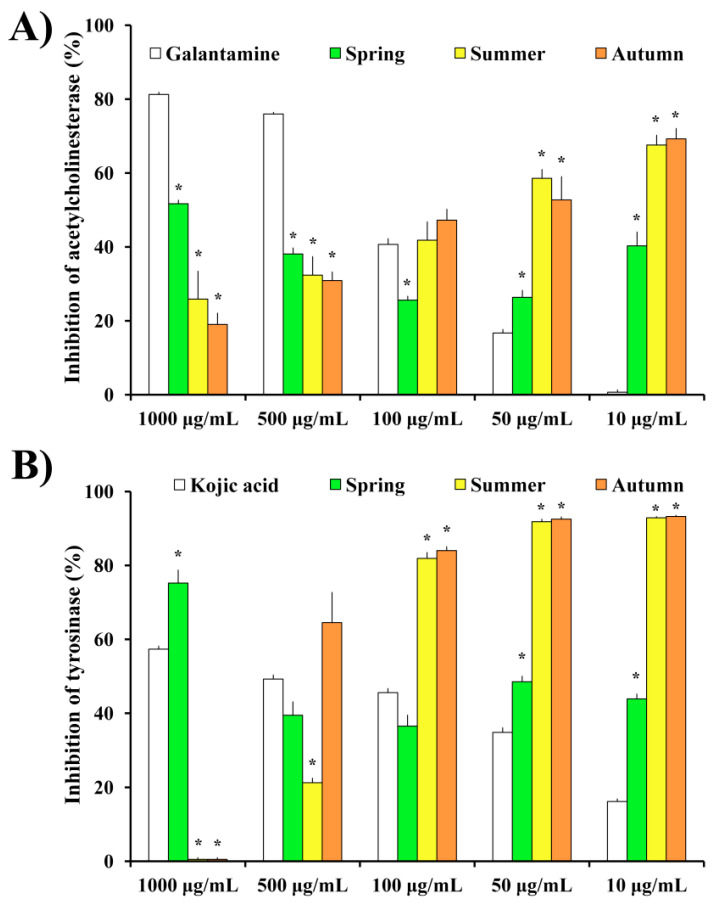
The inhibitory potential of *H. cupressiforme* ethyl acetate extracts from different seasonal aspects (spring, summer, and autumn) on (**A**) AChE and (**B**) mTyr. The results are expressed as the mean ± standard error (* *p* < 0.05 different seasonal aspects vs. corresponding standard substances).

The results obtained regarding the AChE and mushroom tyrosinase (mTyr) inhibitory activities are presented in Figure 5A,B, respectively.

In terms of AChE inhibition, the extracts exhibited moderate activities at the highest tested concentration, while on the lower concentrations the inhibition percentages were high and statistically significant in comparison with the positive control, galantamine (Figure 5A). Interestingly, while the control displayed a concentration-dependent inhibition of AChE, the inhibition values for investigated moss extracts increased proportionally with the decreasing of sample concentration. Thus, the highest inhibition value of AChE was noted at the concentration of 10 μg/mL for both summer and autumn samples. Regarding investigated seasonal aspects, the trends of enzyme inhibition are similar for both summer and autumn aspects of the tested moss species. Furthermore, the inhibition of mTyr followed the same trend as the previously described inhibition of AChE, with a notable increase of the enzyme inhibition percentage as the concentration of moss extract decreased (Figure 5B). Moss extracts at concentrations of 100, 50, and 10 μg/mL exhibited higher inhibition percentages than those of the standard inhibitor substance, kojic acid. Regarding seasonal aspects, summer and autumn aspects displayed a similar trend towards the inhibition of mTyr at all investigated concentrations, although at 500 μg/mL extract from autumn season exhibited considerably higher (but not statistically significant) inhibition percentage than the summer aspect (64.5 vs. 21.2% for autumn and summer, respectively).

### 2.5. In Silico Molecular Docking

Molecular docking was performed on the 14 compounds that were previously identified as secondary metabolites in *H. cupressiforme* extract using LC-MS [10]. AutoDock 4.2.6 simulation was employed to define the binding affinity and potential binding mode of the compounds that could explain AChE and tyrosinase (mushroom and human—hTyr) inhibitory activities. Control docking procedure was performed using the co-crystallized control ligands in order to validate the docking simulation, while galantamine and kojic acid were employed as the experimentally used standard inhibitors towards the AChE and mTyr enzymes, respectively. 

With the aim to evaluate whether the docking protocol and parameters used in this simulation can predict the native conformations of the investigated compounds, validation was carried out by re-docking the AChE and mTyr receptors with ligands bis(7)-tacrine and tropolone, respectively. Precisely, the root mean square deviation (RMSD) value of the re-docked bis(7)-tacrine was found to be 0.83 Å, while for the tropolone the RMSD value was 1.30 Å, suggesting the efficiency and validity of the docking protocol used in the present study. Generally, the docking parameters are considered to be acceptable if the RMSD value of the re-docked ligand, with respect to the crystallized one, is less or equal to 2 Å.

**Figure 6:**
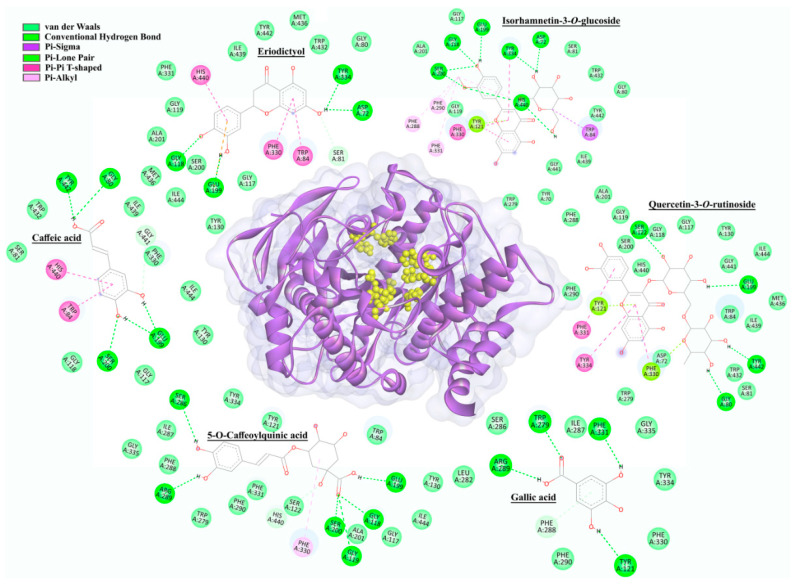
The 2D intermolecular interactions of selected representatives within the active site (marked with yellow) of AChE (2CKM).

**Figure 7:**
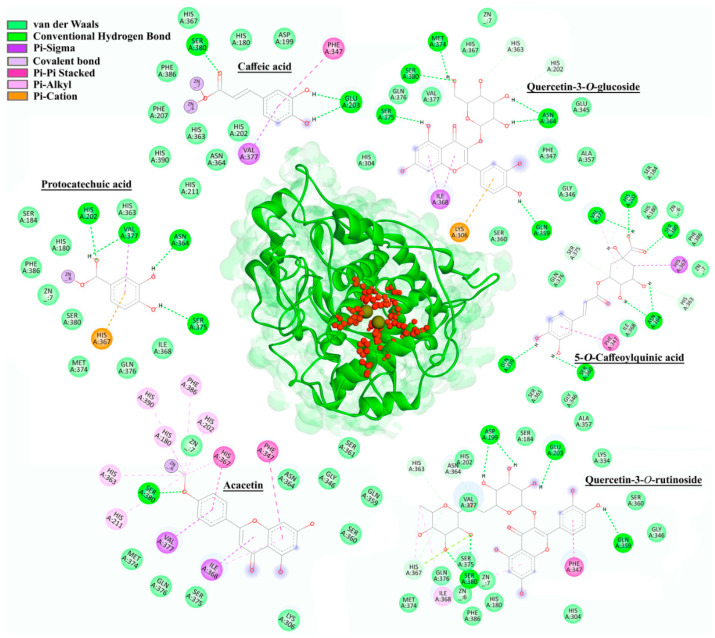
The 2D intermolecular interactions of selected representatives within the active site (marked with red) of hTyr.

Moreover, it should be noticed that all compounds docked on AChE, mTyr, and hTyr active sites were classified into six groups, according to their chemical structures. Within each of these groups, the compounds with the best docking scores were selected, and their interactions with the corresponding enzymes are presented in Figure 6 (AChE) and Figure 7 (hTyr). In addition, docking energies, inhibition constants, and ligand efficiencies, together with all the interactions of investigated enzymes with compounds identified in *H. cupressiforme* are given in Tables S2–S4, for AChE, mTyr, and hTyr, respectively.

#### 2.5.1. AchE Docking

Molecular docking results for the six best-ranked representatives from each compound class (defined according to their chemical structure) docked into the active site of AChE from *Torpedo californica* Ayres, 1855 are presented in Figure 6.

Based on docking results presented in Figure 6, it can be observed that compounds selected within each of six groups as best-docked to AChE active site are: eriodictyol (−8.80 kcal/mol), isorhamnetin-3-*O*-glucoside (−7.34 kcal/mol), 5-*O*-caffeoylquinic acid (−5.86 kcal/mol), caffeic acid (−5.32 kcal/mol), quercetin-3-*O*-rutinoside (−4.79 kcal/mol), and gallic acid (−4.67 kcal/mol). The selected compounds are mainly involved in conventional hydrogen, Pi–Pi, and van der Waals interaction types with Ser200 and His440, important members of the catalytic triad of AChE. In addition to the mentioned types of interactions, carbon–hydrogen, Pi–anion, Pi–lone pair, and Pi–alkyl interactions were also realized with Asp72, Gly80, Ser81, Trp84, Gly118, Glu199, Tyr121, Ser122, Trp279, Ser286, Phe288, Arg289, Phe290, Phe330, Phe331, Tyr334, Gly441, and Tyr442 amino acid residues in the AChE enzyme (Table S2). 

Galantamine, used as a positive control in the enzyme inhibition assay, showed a docking score of −8.99 kcal/mol to the active site of AChE. This docking score suggests a higher binding affinity of galantamine in comparison to investigated compounds from *H. cupressiforme*. The network of hydrogen bonding, van der Waals, carbon–hydrogen, and Pi–alkyl interactions was observed between galantamine and amino acids from AChE, precisely with Trp84, Glu199, Phe288, Phe290, Phe330, Phe331, and His440 (Table S2). 

The group of compounds that exhibited the best binding affinity for the enzyme are flavonoids. This group included five compounds—eriodictyol, acacetin, apigenin, naringenin, and kaempferol, with their corresponding docking scores of −8.80, −8.72, −8.59, −8.52, and −8.39 kcal/mol, respectively. All these compounds interacted with His 440, as well as with Asp72, Gly119, Phe330, and Tyr442, while some of these compounds also interacted with Trp84, Gly118, Gly119, Ala201, Trp233, Phe290, and Tyr334 (Table S2).

#### 2.5.2. Tyrosinase (mTyr and hTyr) Docking

Based on the docking results (Table S3), there were certain differences between mTyr and hTyr when it comes to the affinity of the investigated compounds for the active site of an enzyme. Compounds with the highest inhibitory activity against mTyr within each of six groups were quercetin-3-*O*-rutinoside (−7.72 kcal/mol), *p*-coumaric acid (−6.81 kcal/mol), protocatechuic acid (−6.47 kcal/mol), 5-*O*-Caffeoylquinic acid (−6.42 kcal/mol), apigenin (−5.73 kcal/mol), and isorhamnetin 3-*O*-glucoside (−5.71 kcal/mol). The main types of interactions between the selected compounds and enzyme residues were conventional hydrogen, carbon–hydrogen, alkyl, Pi–Pi, Pi–sigma, Pi–sulfur, Pi–alkyl, and van der Waals interactions. In addition to amino acids from the mTyr active site (His61, His85, His94, His259, His263, and His296), the following residues also participated in the building of mentioned interactions: His244, Val248, Glu256, Met257, Asn260, Thr261, Phe264, Arg268, Pro277, Met280, Gly281, Ser282, Val283, and Ala286. Kojic acid, as a positive standard used in the enzyme inhibition assay, had a docking score of −4.52 kcal/mol, which was weaker than all the compounds investigated in this study. These results also showed that some of the compounds formed Pi–alkyl interactions with Cu400 and Cu401 (Table S3). 

Since there is no currently available crystal structure of hTyr on PDB, a homology modeling-based approach was used to predict the 3D structure of this enzyme. Due to the highest sequence identity with hTyr (43.32%), the crystal structure of human tyrosinase-related protein 1 (hTyrP1, PDB ID: 5M8L) was used as the protein template. The full sequence of hTyr (P14679) was obtained from the UniProt database. Zinc ions were kept in the constructed homology model of hTyr.

Molecular docking results for the six best-ranked representatives from each compound class docked into the active site of hTyr model structure are presented in Figure 7. Although docking simulations were performed for both mTyr and hTyr, only the results for hTyr are presented, due to its greater significance in terms of potential application.

Based on the results of docking simulations performed on hTyr, the following representatives of the six groups of compounds were selected as the best-ranked: caffeic acid (−7.02 kcal/mol), protocatechuic acid (−6.94 kcal/mol), 5-*O*-caffeoylquinic acid (−6.70 kcal/mol), acacetin (−6.27 kcal/mol), quercetin-3-*O*-glucoside (−5.75 kcal/mol), and quercetin 3-*O*-rutinoside (−5.65 kcal/mol). The main binding forces between the compounds and hTyr were conventional hydrogen, carbon–hydrogen, alkyl, Pi–Pi, Pi–sigma, Pi–alkyl, Pi–cation, and van der Waals interactions. Amino acids involved in the interactions comprised of residues in the active site (His180, His202, His211, His363, His367, and His390) as well as other residues, including Asp199, Glu203, Lys306, Phe347, Gln359, Asn364, Ile368, Met374, Ser375, Val377, Ser380, and Phe386. The binding affinity of kojic acid to hTyr was measured with a docking score of −4.26 kcal/mol, which was weaker than that of mTyr (−4.52 kcal/mol). Compounds caffeic acid, protocatechuic acid, and acacetin were also observed to interact with zinc ions (Zn6 for acacetin and protocatechuic acid, while caffeic acid interacted with both Zn6 and Zn7, Figure 7).

## 3. Discussion

In the search for novel, naturally occurring bioactive compounds from plants, bryophytes, especially mosses, have been relatively overlooked for a quite long time. However, due to the remarkable structural and chemical diversity of compounds identified in mosses, much more attention has been given to these plants recently. In our previously published paper [10], different extracts of *H. cupressiforme* samples collected during the spring season have been (bio)chemically and biologically evaluated. The results of that investigation served as the starting point in this research since promising biological potential has been observed. Additionally, knowing that the content of secondary metabolites in plants can vary depending on the season, in the present paper, the ethyl acetate extract of moss *H. cupressiforme* (previously proved as biologically most prominent) was chemically characterized throughout three different seasons (spring, summer, and autumn) and screened for its antioxidant, anti-neuroinflammatory, anti-neurodegenerative, and neuroprotective potential. Because winter is generally considered as a dormant season for plants, including some bryophytes [14,17] the winter aspect of moss *H. cupressiforme* was not included in the present study.

All moss samples (parts of the huge mats covering rock outcrops) were collected at the same location and from the same population (Vršačke Planine Mts., Serbia), while the regeneration is documented by the absence of sex organs as vegetative (implying to same genetic structure). Thus, the variation in extract secondary metabolite content can only be attributed to seasonal climatic characteristics, such as fluctuations in temperature, precipitation, air humidity, and/or duration and intensity of sun radiation. The results presented in Figure 1A revealed that moss *H. cupressiforme* produced the highest content of all investigated secondary metabolites (TPC, TPAC, TFC, TFlC, and TTC) during the summer season. These compounds are likely elevated during summer to enable the moss to cope with abiotic stress such as high temperatures and droughts. In the literature, however, contrasting tolerance patterns were observed on a seasonal level among different bryophytes. While in some studies the majority of protective substances such as polyphenols were exhibited during the summer, in others, the concentration of phenolic substances was the highest in spring and gradually decreased during the season [12,18]. These data suggest that bryophytes respond species-specifically to different seasonal conditions. Additionally, the assumption on synergisms and antagonisms should be considered as well. Bearing in mind all previously stated, studies about seasonal variation in moss chemical composition are essential to determine the season that is the most productive for a particular species of interest. Moreover, it can be regionally depending. For *H. cupressiforme*, based on the results from this study, the season with the highest content of secondary metabolites of interest locally proved to be summer. 

Since different classes of polyphenolic compounds with antioxidant properties have been identified in *H. cupressiforme*, our next goal was to examine the antioxidant potential of this moss. The β-carotene assay was used as a model system, where the capacity of extracts for the inhibition of lipid peroxidation was evaluated. The results of the β-carotene assay (Figure 1B) revealed that all three seasonal aspects of moss *H. cupressiforme* performed the same or even better activities than natural antioxidant ascorbic acid. Among investigated seasons, the summer aspect exhibited the best activity at all tested concentrations, except the lowest. The probable explanation for the highest activity in summer aspect is that exposure to higher temperatures during the summer is linked to a higher need for antioxidant protection in tested moss. This is in accordance with the existing literature data, where it can be found that mosses exhibit higher antioxidant enzymatic activity during the summer [19]. Nevertheless, it should be noted that this is the first study that evaluated the seasonal change of antioxidant activity in mosses by using the β-carotene bleaching assay. 

The antioxidant and anti-neuroinflammatory potential of examined moss extracts was further examined on BV2 microglial cells, where oxidative stress induced by H_2_O_2_ served as the main trigger of inflammation. The treatment of BV2 cells with H_2_O_2_ significantly reduced cell viability and increased the oxidative stress in cells through the production of ROS. The same observations have been reported in several studies where BV2 cells were exposed to H_2_O_2_ and activated by direct oxidation, leading to the subsequent production of ROS [20,21,22]. Although ROS are essential for maintaining neuronal cell functions, excessive oxidative stress can cause protein oxidation and lipid peroxidation, thus promoting neuronal damage and degeneration. Given that moss extracts normalized the viability of H_2_O_2_-stimulated microglia cells and diminished their production of ROS (Figure 2), bringing them to the level of untreated controls, investigated moss extracts can be considered as potent antioxidants and anti-neuroinflammatory agents. The observed antioxidant activity in cells could be mediated through the capacity of extracts to protect against lipid peroxidation, such as previously shown in β-carotene bleaching assay, using a cell-free model system. Extracts from both investigated seasons (summer and autumn) performed similar activities, without significant differences between them, suggesting that both aspects of moss *H. cupressiforme* might carry therapeutic potential against neurodegenerative diseases that have been associated with oxidative stress [23]. 

Another potent trigger for the activation of BV2 cells and secretion of inflammatory mediators is LPS, an endotoxin produced by Gram-negative bacteria. In our previous study, we already established the potential of *H. cupressiforme* extracts from the spring season to act as anti-neuroinflammatory agents against BV2 cells activated by LPS [10]. Therefore, the goal of the present study was to find out the potential of extracts from summer and autumn seasons to act as anti-inflammatory agents in the same model system. The results presented in Figure 3 have shown that LPS stimulation significantly decreased the metabolic activity of BV2 cells and increased the production of inflammatory cytokines (TNF-α and IL-6) as well as inflammatory mediators (ROS and NO), causing inflammation as a protective response of the immune system. Sometimes, this response may be so strong and uncontrolled that it leads to chronic inflammation, which is associated with the development of different neurodegenerative pathologies [24]. Therefore, it is very important to limit the production of inflammatory cytokines and mediators by microglia and to keep the inflammatory response under control. In this study, we found that *H. cupressiforme* extract reduced the production of cytokines and mediators by LPS-activated microglia cells. Additionally, extracts normalized the metabolic activity of LPS-treated cells, recovering and bringing them to the level of untreated, control cells. Both investigated seasons (summer and autumn) exhibited significant anti-neuroinflammatory activities, which is additional evidence of *H. cupressiforme*’s applicability in the prevention and treatment of neuroinflammatory and neurodegenerative disorders.

The neuroprotective potential of *H. cupressiforme* extracts was evaluated using the microglial culture supernatant transfer model. In this model system, we examined how soluble molecules released by BV2 microglial cells (LPS-stimulated and treated with investigated moss extract) affect neuronal cells and their metabolic activity. As observed in previous studies [25,26], supernatants from LPS-stimulated BV2 microglial cells have induced toxicity to SH-SY5Y neuronal cells. However, treatment of BV2 cells with moss extracts upon LPS stimulation significantly increased metabolic activity in SH-SY5Y cells, bringing them to the level of control cells (Figure 4). These results suggest that summer and autumn aspects of moss *H. cupressiforme* possess a significant capacity to protect neuronal cells against neurotoxicity induced by LPS. Therefore, extracts of moss *H. cupressiforme* have a promising potential for preventing and treating neurodegenerative diseases associated with excessive microglial activation, neuroinflammation, and subsequent neurotoxic consequences.

With the aim to examine the neuroprotective effects of *H. cupressiforme* extracts, we also evaluated AChE and tyrosinase inhibitory potential. AChE is an enzyme that catalyzes the hydrolysis of the neurotransmitter acetylcholine to acetate and choline. Although AChE is essential for the proper functioning of the nervous system, increased activity of this enzyme can lead to problems in synaptic integrity, neurite outgrowth, and neurodevelopment [27]. Moreover, low levels of acetylcholine in the synapses due to increased AChE activity have been linked to memory loss commonly seen in Alzheimer’s disease (AD) [28]. Furthermore, we investigated the inhibition of tyrosinase, an enzyme related to another neurodegenerative disease, Parkinson’s disease (PD). It has been reported that tyrosinase is a key enzyme involved in the formation of neuromelanin in the central nervous system [29]. As the accumulation of neuromelanin is associated with the damage of neurons observed in PD, the inhibition of tyrosinase is a promising approach for PD treatment [30]. In the present study, AChE and mTyr inhibitory potential of *H. cupresifforme* from summer and autumn seasons were investigated, while the inhibitory potential for the spring aspect was published recently [10]. The obtained results (Figure 5) show that extracts from summer and autumn seasons strongly inhibit both AChE and mTyr, performing better activities at the lower concentrations. Interestingly, in both AChE and mTyr inhibition assays, opposite dose-responses were observed. These phenomena can be addressed to different intermolecular interactions at higher concentrations, which finally decreased the total amount of available molecules for the interaction with enzymes. Consequently, the inhibitory potential of extracts is reduced at high concentrations, while prominent activates are observed at lower concentrations.

Molecular docking studies of the 14 compounds previously identified in *H. cupressiforme* ethyl acetate extract [10] to enzymes AChE and tyrosinase have been performed. The 3D structure of AChE is evolutionary conserved, so AChE’s from different species are generally very similar [31]. Thus, we expect that the structure of AChE employed for the docking simulation in this study is comparable to the human and should possess identical properties of the active site. Accordingly, the obtained results for *Torpedo californica* AChE could be translated to human AChE. On the other hand, due to differences in activities and substrate specificities between mTyr and hTyr, compounds that are potent inhibitors of mTyr are not always effective against hTyr, as previously reported [32]. As the final goal of this research was to define the most efficient secondary metabolites from moss extracts in terms of potential human application, the docking on hTyr was also performed.

Regarding AChE (Figure 6), it has been observed that all investigated compounds bind in close proximity of the AChE active site, which includes the catalytic site (Ser200, His 440, and Glu327) as well as many additional subsites also important for the catalytic process. In the anionic subsite (Trp84, Tyr130, Tyr330, and Phe331) the choline moiety of the substrate is bound and positioned for hydrolysis. The acyl pocket (Phe288 and Phe290) binds the acetyl group of the substrate, while the oxyanion hole (Gly118, Gly119, and Ala 201) is included in the stabilization of the substrate transition state [33]. Among the investigated ligands, the strongest binding affinity toward AChE was obtained for eryodictiol and, generally, for all flavonoids. These compounds have shown interactions (Pi–Pi and van der Waals) with important amino acid residues, His440 and Ser200, within the catalytic site of AChE. In addition, flavonoids realized several interactions with prominent amino acid residues of an anionic subsite of AChE, namely Pi–Pi interactions with Trp84 and Tyr330, as well as van der Waals interactions with Tyr130 and Phe331. These interactions are similar to those of galantamine, the standard substance used for AChE inhibition. In light of present results, flavonoids identified in *H. cupressiforme* exhibited a great inhibitory potential towards AChE, which has been reported in the literature as well [34]. 

Docking simulations for both mTyr and hTyr confirmed that all previously identified compounds in *H. cupressiforme* extracts bind to the active site of tyrosinase more strongly than standard, kojic acid. This is in agreement with in vitro tyrosinase inhibition assay where the results suggested that moss extracts have higher inhibitory potential against the enzyme in comparison to kojic acid, especially at lower concentrations, due to a smaller chance for intermolecular formations. The best docking score for the mTyr was obtained for a flavonoid glycoside (quercetin-3-*O*-rutinoside), while hydroxycinnamic acid derivative (caffeic acid), showed the highest binding affinity toward hTyr. This suggests that compounds similar to caffeic acid in structure might be effective in the inhibition of the human enzyme. Namely, hydroxycinnamic acid derivatives have been previously reported as successful compounds in the inhibition of human tyrosinase [35,36]. Finally, as there is evidence in the literature about the diverse inhibition profiles of hTyr and mTyr [32], the establishment of intercorrelation between available experimental models (not human) and in silico data is a useful approach for the translation of in silico results obtained for human proteins into the expected experimental results.

## 4. Materials and Methods

### 4.1. Plant Material

Specimens of moss *Hypnum cupressiforme* Hedw. (summer and autumn aspects) were collected in the Vršačke Planine Mts., Serbia (N45.128208, E21329945, 370 m a.s.l.) and prepared as previously described for the spring aspect [10]. Moss material was sampled from the siliceous rock outcrops within the forest openings from the same population in different seasons according to the local climatic conditions adjusted by average values for the seasonal mean temperatures and precipitates. Thus, the collection dates were chosen as follows: spring-time (11 May 2019), summer-time (20 August 2020), and autumn-time (1 December 2020) (leg./det. M. S. Sabovljevic and A. D. Sabovljevic, vouchers BEOU bryo collection s/n). Permission for the plant material collection was provided by the Serbian Ministry of Environment (No. 353-01-798/2020-04).

Mosses were further placed in paper bags and kept at room temperature. The room-dried and cleaned materials (i.e., green tips with no older parts and substrate remnants) were then lyophilized and ready for extraction. Moss material (5 g dry weight) was ground into small pieces in a cylindrical crusher and extracted with 100 mL of ethyl acetate for 10 h, using Soxhlet apparatus. The extracts were concentrated under reduced pressure with a rotary evaporator at 40 °C (Buchi R-210 Rotavapor System, Marshall Scientific, Hampton, NH, USA) and finally stored in dark at 4 °C. 

### 4.2. Determination of Selected Classes of Secondary Metabolites

Total phenolic (TPC), phenolic acid (TPAC), flavonoid (TFC), flavonol (TFlC), and triterpenoid contents (TTC) were measured as described previously [10] using Multiskan Sky Thermo Scientific microtiter plate reader, Vantaa, Finland. The phenolic content of extracts was calculated from the gallic acid curve equation and expressed as milligrams of gallic acid equivalents per gram of dry extract (mg GAE/g dry extract). The phenolic acid content of extracts was calculated from the curve equation of caffeic acid in 50% ethanol and expressed as milligrams of caffeic acid equivalents per gram of dry extract (mg CAE/g dry extract). Flavonoid and flavonol contents of extracts were calculated from the curve equation of quercetin and expressed as milligrams of quercetin equivalents per gram of dry extract (mg QE/g dry extract). Triterpenoid content of extracts was calculated from the ursolic acid curve equation and expressed as milligrams of ursolic acid equivalents per gram of dry extract (mg UAE/g dry extract).

### 4.3. Antioxidant Activity

Linoleic acid/β-carotene bleaching assay was performed according to a method [37] described previously [10]. The absorbance was measured using the Multiskan Sky Thermo Scientific Microtiter plate reader, Vantaa, Finland. The results are expressed as the percentage of β-carotene bleaching inhibition. Ascorbic acid (AA) was used as an antioxidant standard (positive control). The concentrations of investigated moss extracts and standard were 1000, 500, 100, 50, and 10 μg/mL.

### 4.4. Anti-Neuroinflammatory Activity

#### 4.4.1. Cell Culture

Human embryonic lung fibroblast cell line (MRC-5), murine microglial cell line (BV2), and human neurons (SH-SY5Y) were used in the present study. The cells were obtained from American Tissue Culture Collection (ATCC, Manassas, VA, USA).

All cells were cultivated in RPMI-1640 and supplemented with 10% FBS, 1% glucose, and 1% antibiotics (penicillin and streptomycin). The cells were maintained at 37 °C in a humidified atmosphere containing 5% CO_2_. Confluent cells were seeded in a 96-well microplate. After 24 h of cell incubation, 100 µL of medium containing investigated moss extract was added to each well of the microplate. Untreated cells were used as control. The treatment concentration (10 µg/mL) was obtained by serial dilution of the stock solution (prepared in DMSO) with full medium, thus the concentration of DMSO decreased continuously and was under 0.05%.

#### 4.4.2. Stimulation of BV2 Microglial Cells

Murine microglial BV2 cells were seeded into 96-well plates at a concentration of 10^4^ cells per well and allowed to grow for 24 h. After reaching confluency, the following stimuli were added to the cells at concentrations determined in preliminary experiments: LPS at 10 μg/mL and H_2_O_2_ at 30 μM, while the investigated moss extracts were added at final concentration of 10 μg/mL of cell culture. Precisely, 100 µL of LPS or H_2_O_2_ containing the investigated extracts was added to each well. The incubation was continued for an additional 48 h after which the cells were subjected to MTT, NBT, and Griess assays.

#### 4.4.3. Microglial Culture Supernatant Transfer Model

To test the neurotoxic effects of activated microglia, human neurons SH-SY5Y were plated in 96-well plates at a concentration of 2 × 10^4^ cells per well in full medium. After 24 h incubation, supernatants (100 μL) of LPS-stimulated BV2 cells treated with moss extracts were added to a 96-well microplate seeded with SH-SY5Y neurons, and incubation continued for another 24 h. The metabolic activity of the SH-SY5Y cells was then measured using the MTT assay.

#### 4.4.4. MTT Assay

The effects of moss extracts on cell metabolic activity were evaluated utilizing the MTT assay [38] as described in our previous study [10]. The absorbance of the reduced MTT was measured at 540 nm using a microplate reader (LKB 5060-006, LKB Instruments, Vienna, Austria). The results are presented as the metabolic viability of cells, calculated as the ratio between the absorbance of treated cells and the absorbance of the untreated control cells multiplied by 100.

#### 4.4.5. NBT Assay

The influence of investigated moss extracts on the production of superoxide anion radical (O_2_^−^·) by BV2 cells was evaluated via NBT assay [39]. The experimental protocol was described with details in our recently published study [10]. The absorbance of the generated formazan solution was measured at 540 nm using a microplate reader LKB 5060–006, LKB Instruments, Vienna, Austria. The results are presented as the mean values of the ROS index, calculated as the ratio between the absorbance of treated cells and the untreated control cells.

#### 4.4.6. Griess Assay

The determination of the nitric oxide (NO) production was performed by using the spectrophotometric method based on the Griess reaction [40] which is with details described in the following study [9]. The absorbance of the solution was measured at 540 nm using a LKB 5060–006, LKB Instruments, Vienna, Austria LKB 5060–006, LKB Instruments, Vienna, Austria microplate reader. The results are calculated from the nitrite standard curve and presented as nitrite concentration (µM) which is equivalent to the NO concentration in the samples.

#### 4.4.7. Measurement of Cytokine Levels in Cell Supernatants

Supernatants of LPS-stimulated BV2 cells from the experiments above were collected and concentrations of cytokines IL-6 and TNF-α were determined. Quantification of cytokines was carried out using enzyme-linked immunosorbent assay (ELISA) kits according to the manufacturer’s suggestions (R&D Systems). The results are expressed in pg/mL.

### 4.5. Anti-Neurodegenerative Activity

AChE [41] and tyrosinase [42] inhibitory activity assays were performed according to a protocol described previously [10]. Moss extracts and standards (galantamine and kojic acid) were investigated at following concentrations 1000, 500, 100, 50, and 10 μg/mL using Multiskan Sky Thermo Scientific Microtiter plate reader, Vantaa, Finland. The results are presented as a percentage of enzyme inhibition in comparison to the corresponding standard substances (galantamine and kojic acid for AChE and tyrosinase, respectively).

### 4.6. Molecular Docking

Molecular docking studies were carried out in order to examine the binding modes of AChE and tyrosinase with 14 compounds previously identified in *H. cupressiforme* extracts by LC-MS [10]. All investigated structures of proteins were retrieved in pdb format from the RCSB Protein Data Bank database of biological macromolecules, or when the corresponding crystal structure was not available, a homology model was constructed using SWISS-MODEL server [43]. The proteins were prepared for molecular docking by removing co-crystallized ligands, water molecules, and cofactors (BIOVIA Discovery Studio 2021 [44]. Missing amino acid residues were added using Modeller 10.1 [45]. The AutoDockTools (ADT [46]) graphical interface was used to add polar hydrogen, adjust protonation states of histidine amino acid residues, and to add Kollman charges to proteins.

AChE from *Torpedo californica* was chosen for molecular docking studies due to the previously confirmed homology with AChE from *Electrophorus electricus* [47], and better resolution of the *Torpedo californica* AChE. The three-dimensional (3D) crystal structure of *Torpedo californica* AChE complexed with bis(7)-tacrine (PDB ID: 2CKM) was retrieved with a resolution of 2.15 Å. 

Tyrosinase from *Agaricus bisporus* was selected as the protein model for the present study since the mushroom tyrosinase (mTyr) enzyme was used in the inhibition assay. The 3D crystal structure of tyrosinase from *Agaricus bisporus* in complex with the inhibitor tropolone (PDB ID: 2Y9X) was retrieved with a resolution of 2.78 Å. Additionally, the human tyrosinase (hTyr) 3D structure has been predicted based on amino acid sequence obtained from UniProt and homology modeling was performed on SWISS-MODEL. 

Ligand structures were retrieved from the PubChem Compound database in sdf formats and energy minimized using the UFF (Universal Force Field) implemented in the Avogadro software [48]. Gasteiger charges were assigned to the ligands using the ADT graphical interface.

#### Docking Procedure

Docking studies were carried out using AutoDock software (version 4.2.6 [46]) which is equipped with ADT graphical interface. The size of the grid box was set to be 60 Å × 60 Å × 60 Å in the x, y, and z directions with a default grid point spacing of 0.375 Å. The center of the box was determined by the coordinates of the amino acids in the active site of an enzyme. Precisely, the exact coordinates used for AChE were X = 11.513, Y = 67.693, Z = 62.518; the coordinates for mTyr were as following X = −10.044, Y = −28.706, Z = −43.443; and the coordinates for hTyr were X = 36.339, Y = 140.689, Z = 215.645. The Lamarckian genetic algorithm (LGA) was employed to generate ligand orientation within the active site. Docking of each ligand to protein was performed with 200 iterations. The conformations were manually checked and the ones with the lowest energies in the highest numbered population size of cluster were selected for further analysis and representation. Visualization and analysis of the docking results were carried out by means of ADT and BIOVIA Discovery Studio 2021.

Before screening the compounds of interest, the docking protocol was validated by re-docking the ligands extracted from the crystal structures of AChE and tyrosinase (bis(7)-tacrine and tropolone, respectively). The docking protocol that is previously described was used in the re-docking process. The RMSD of all heavy atoms between the docked and crystal conformations of ligands were computed.

### 4.7. Statistical Analysis

The statistical analysis of the data was performed using the Statistical Package for Social Sciences program (SPSS) (IBM SPSS Statistics for Windows, Version 25.0., IBM Corporation, Armonk, NY, USA). Statistical evaluation was performed by Independent Samples *t*-test, while the minimum probability value taken as statistically significant was *p* < 0.05. All measurements were carried out at least in triplicate, and results were presented as mean ± standard error.

## 5. Conclusions

The results of the present study demonstrate a significant variation in the total amount of secondary metabolites in moss *H. cupressiforme* extracts throughout different seasons, with the highest content of all investigated compounds produced during the summer season. Accordingly, the moss from the summer season also expressed the highest antioxidant activity, as evaluated by β-carotene assay. Extracts from summer and autumn seasons have shown the ability to reduce oxidative and inflammatory stresses in murine BV2 microglial cells and human SHSY-5Y neuronal cells by inhibiting the production of inflammatory mediators such as ROS, NO, IL-6, and TNF-α. This evidence of anti-neuroinflammatory and neuroprotective properties of *H. cupressiforme* was further confirmed by examination of the inhibitory potential toward AChE and tyrosinase, where extracts at low concentrations exhibited better inhibition rates than corresponding standard substances. Finally, docking simulations revealed that, among the compounds identified in the extract, flavonoids exhibited the strongest inhibition potential by making interactions with the active site of AChE, while hydroxycinnamic acid derivatives showed the best affinity toward the hTyr. Finally, the results from the present study significantly enlighten the traditional usage of *H. cupressiforme*, establishing its therapeutic efficacy as an anti-inflammatory source, and revealing its neuroprotective properties. Special attention should be addressed to seasonal variation of *H. cupressiforme* secondary metabolites, where the summer aspect was found as the most prominent.

## Supplementary Materials

The following are available online at https://www.mdpi.com/article/10.3390/plants11010123/s1, Table S1: Chemical characterization of examined seasonal aspects of moss *H. cupressiforme* ethyl acetate extracts. Table S2: Docking results and interactions between the compounds identified in *H. cupressiforme* docked into AChE active site. Table S3: Docking results and interactions between the compounds identified in *H. cupressiforme* docked into mushroom tyrosinase active site. Table S4: Docking results and interactions between the compounds identified in *H. cupressiforme* docked into human tyrosinase active site.
