# Supplementary material for: The Influence of Seasonality on Secondary Metabolite Profiles and Neuroprotective Activities of Moss Hypnum cupressiforme Extracts: In Vitro and In Silico Study
Source: Plants (Basel). 2022 Jan 1;11(1):123. doi: 10.3390/plants11010123 (PMC8747716; doi:10.3390/plants11010123)
Supplement: Supplementary file 1 [file plants-11-00123-s001.zip › plants-1537198-supplementary.pdf]

Supplementary material

The Influence of Seasonality on Secondary Metabolite Profiles and Neuroprotective Activities of Moss *Hypnum cupressiforme* Extracts: In Vitro and In Silico Study

Tanja M. Lunić[1], Marija R. Mandić[1], Mariana M. Oalđe Pavlović[2], Aneta D. Sabovljević[2], Marko S. Sabovljević[2, 3], Biljana Đ. Božić Nedeljković[1*#], Bojan Đ. Božić[1*#]

[1] Institute of Physiology and Biochemistry "Ivan Djaja", Faculty of Biology, University of Belgrade, 11000 Belgrade, Serbia; lunictanja@hotmail.com (T.L.); mandicmarija5@gmail.com (M.M.); biljana@bio.bg.ac.rs (B.B.N.); bbozic@bio.bg.ac.rs (B.B.)

[2] Institute of Botany and Botanical Garden "Jevremovac", Faculty of Biology, University of Belgrade, 11000 Belgrade, Serbia; marianao@bio.bg.ac.rs (M.O.); aneta@bio.bg.ac.rs (A.S.); marko@bio.bg.ac.rs (M.S.)

[3] Department of Botany, Institute of Biology and Ecology, Faculty of Science, Pavol Jozef Šafárik University in Košice, Mánesova 23, 040 01 Košice, Slovakia

* Correspondence: bbozic@bio.bg.ac.rs; Tel.: +381 11 303 23 56

[#] These authors contributed equally to this study.

Table S1. Chemical characterization of examined seasonal aspects of moss *H. cupressiforme* ethyl acetate extracts.

Seasonal Aspect	TPC (mg GAE/g extract)	TPAC (mg CAE/g extract)	TFC (mg QE/g extract)	TFlC (mg QE/g extract)	TTC (mg UAE/g extract)
**Spring[10]**	15.33 ± 0.95	339.93 ± 14.03	58.86 ± 2.82	14.11 ± 1.33	235.95 ± 4.09*
**Summer**	21.87 ± 1.13*#	661.70 ± 17.14*#	108.21 ± 5.04*#	53.56 ± 3.51*#	256.44 ± 6.88*
**Autumn**	15.12 ± 0.85	357.99 ± 7.52	80.39 ± 4.56#	37.86 ± 1.24#	144.79 ± 1.23

vs. spring; * *vs.* autumn

CAE – caffeic acid equivalents; GAE – gallic acid equivalents; QE – quercetin equivalents; TPAC – total phenolic acid content; TFC – total flavonoid content; TFlC – total flavonol content; TPC – total phenolic content; TTC – total triterpenoid content; UAE – ursolic acid equivalents

Table S2. Docking results and interactions between the compounds identified in *H. cupressiforme* docked into AChE active site.

Compound	ΔG (kcal/mol)	Ki (μM)	Ligand efficiency	Catalytic site interactions*	Interactions out of the catalytic site*
**Galantamine**	-8.99	0.26	-0.43	His440	Trp84, Glu199, Phe288, Phe290, Phe330, Phe331
**Gallic acid**	-4.67	375.08	-0.39	/	Tyr121, Trp279, Phe288, Arg289, Phe331
**Protocatechuic acid**	-4.53	478.55	-0.41	/	Tyr121, Trp279, Phe288, Arg289, Phe330
**5-*O*-Caffeoylquinic acid**	-5.86	51.01	-0.23	Ser200, His440	Gly118, Gly119, Glu199, Phe330, Ser286, Arg289
***p*-Hydroxybenzoic acid**	-4.65	388.37	-0.47	/	Trp279, Ile287, Phe288, Arg289, Phe330
**Caffeic acid**	-5.32	126.94	-0.41	Ser200, His440	Gly80, Trp84, Glu199, Gly441, Tyr442
**Quercetin 3-*O*-rutinoside**	-4.79	310.25	-0.11	/	Gly80, Tyr121, Ser122, Glu199, Phe330, Phe331, Tyr334, Tyr442
**p-Coumaric acid**	-5.05	198.38	-0.42	Ser200	Gly80, Glu199, Tyr442
**Quercetin-3-*O*-glucoside**	-6.76	11.12	-0.20	His440	Asp72, Trp84, Tyr121, Glu199, Phe330
**Isorhamnetin-3-*O*-glucoside**	-7.34	4.20	-0.22	Ser200, His440	Asp72, Trp84, Gly118, Tyr121, Glu199, Phe288, Phe290, Phe330, Phe331, Tyr334
**Eriodictyol**	-8.80	0.35	-0.42	His440	Asp72, Ser81, Trp84, Gly118, Glu199, Phe330, Tyr334
**Apigenin**	-8.59	0.51	-0.43	His440	Asp72, Ser81, Gly118, Phe330, Tyr334, Tyr442
**Naringenin**	-8.52	0.57	-0.43	His440	Asp72, Trp84, Gly118, Gly119, Phe330, Tyr334
**Kaempferol**	-8.39	0.71	-0.40	His440	Asp72, Gly119, Phe330, Tyr442
**Acacetin**	-8.58	0.51	-0.41	His440	Asp72, Gly118, Gly119, Ala201, Trp233, Phe290, Phe330, Tyr442

*Van der Waals interactions are not included in this table.

Table S3. Docking results and interactions between the compounds identified in *H. cupressiforme* docked into mushroom tyrosinase active site.

Compound	ΔG (kcal/mol)	Ki (μM)	Ligand efficiency	Interaction with Cu^{+2}*	Active site interactions*	Interactions out of the active site*
**Kojic acid**	-4.52	485.01	-0.45	/	His85	Cys83, Asn320, Glu322
**Gallic acid**	-6.14	31.36	-0.51	Cu401	His85, His263	Asn260, Phe264, Met280, Val283, Ala286
**Protocatechuic acid**	-6.47	18.00	-0.59	Cu401	His61, His85, His263	Asn260, Met280, Ser282, Val283, Ala286
**5-*O*-Caffeoylquinic acid**	-6.42	19.67	-0.26	/	His85, His263	Met257, Asn260, Thr261, Met280, Gly281, Val283
***p*-Hydroxybenzoic acid**	-6.30	24.28	-0.63	Cu401	His263	Met280, Gly281, Val283, Ala286
**Caffeic acid**	-5.71	65.68	-0.44	/	/	Val283
**Quercetin 3-*O*-rutinoside**	-7.72	2.21	-0.18	Cu401	His61, His263	His244, Val248, Met257, Asn260, Thr261, Phe264, Arg268, Val283, Ala286
**p-Coumaric acid**	-6.72	11.86	-0.56	/	His263	Gly281, Val283
**Quercetin-3-*O*-glucoside**	-5.67	69.73	-0.17	/	/	His244, Val248, Met257, Asn260, Thr261, Phe264, Arg268, Gly281
**Isorhamnetin-3-*O*-glucoside**	-5.71	64.91	-0.17	/	His85	His244, Val248, Glu256, Thr261, Phe264, Arg268, Gly281, Val283
**Eriodictyol**	-5.29	133.42	-0.25	/	His263	Asn260, Arg268, Pro277, Val283
**Apigenin**	-5.73	63.52	-0.29	/	His263	Asn260, Arg268, Pro277, Ser282, Val283
**Naringenin**	-5.45	100.64	-0.27	/	His263	Asn260, Arg268, Pro277, Val283
**Kaempferol**	-5.26	139.28	-0.25	Cu400, Cu401	His85, His259, His263	Val248, Glu256, Met280, Val283, Ala286
**Acacetin**	-5.39	111.71	-0.26	Cu401	His85, His259, His263	Val248, Met280, Ser282, Val283, Ala286

*Van der Waals interactions are not included in this table.

Table S4. Docking results and interactions between the compounds identified in *H. cupressiforme* docked into human tyrosinase active site.

Compound	ΔG (kcal/mol)	Ki (μM)	Ligand efficiency	Interaction with Zn^{+2}*	Active site interactions*	Interactions out of the active site*
**Kojic acid**	-4.26	748.3	-0.43	/	His367	Asn364, Met374, Val377, Ser380
**Gallic acid**	-6.72	11.84	-0.56	Zn6, Zn7	His367	Asn364, Met374, Ser375, Val377
**Protocatechuic acid**	-6.94	8.19	-0.63	Zn6	His202, His367	Asn364, Ser375, Val377
**5-*O*-Caffeoylquinic acid**	-6.70	12.29	-0.27	/	His202, His363, His367	Phe347, Gln359, Ser360, Asn364, Ser375, Val377, Ser380
***p*-Hydroxybenzoic acid**	-6.77	10.93	-0.68	Zn6	His202, His367	Ser375, Val377
**Caffeic acid**	-7.02	7.17	-0.54	Zn6, Zn7	/	Glu203, Phe347, Val377, Ser380
**Quercetin 3-*O*-rutinoside**	-5.65	72.49	-0.13	/	His363, His367	Asp199, Glu203, Phe347, Gln359, Asn364, Ile368, Ser380
**p-Coumaric acid**	-6.76	11.03	-0.56	Zn6, Zn7	His367	Ser375, Val377
**Quercetin-3-*O*-glucoside**	-5.75	61.46	-0.17	/	His202, His363	Lys306, Gln359, Asn364, Ile368, Met374, Ser375, Ser380
**Isorhamnetin-3-*O*-glucoside**	-5.59	80.08	-0.16	/	His202, His363	His304, Lys306, Ala357, Gln359, Asn364, Ile368, Ser375, Ser380
**Eriodictyol**	-5.92	45.71	-0.28	/	His367	Glu203, Phe347, Met374, Val377, Ser380
**Apigenin**	-5.66	71.28	-0.28	Zn6	His367	Glu203, Phe347, Val377
**Naringenin**	-5.68	68.70	-0.28	Zn6	His367	Glu203, Phe347, Val377
**Kaempferol**	-6.00	40.00	-0.29	/	His367	Ile368, Ser375, Val377
**Acacetin**	-6.27	25.33	-0.30	Zn6	His180, His202, His211, His363, His367, His390	Phe347, Ile368, Val377, Ser380, Phe386

*Van der Waals interactions are not included in this table.
